# Prevalence, Association Relation, and Dynamic Evolution Analysis of Critical Values in Health Checkup in China: A Retrospective Study

**DOI:** 10.3389/fpubh.2021.630356

**Published:** 2021-07-22

**Authors:** Jingfeng Chen, Zhuoqing Wu, Yanan Liu, Lin Wang, Tiantian Li, Yihan Dong, Qian Qin, Suying Ding

**Affiliations:** ^1^Health Management Center, The First Affiliated Hospital of Zhengzhou University, Zhengzhou, China; ^2^Institute of Systems Engineering, Dalian University of Technology, Dalian, China

**Keywords:** critical values in health checkup, prevalence analysis, association relation analysis, dynamic evolution analysis, health managament

## Abstract

**Objective:** The critical values in health checkup play a key role in preventing chronic diseases and different types of cancer. This study aimed to analyze the prevalence, association relation, and dynamic evolution of critical values in health checkups at a large physical examination center in China.

**Methods:** Herein, we chose 33,639 samples of physical examiners from January 2017 to December 2019. After strict exclusion processes, combined with the critical values in health checkup reporting data, 4,721 participants with at least one critical value were included. We first defined a critical value list for laboratory test, imaging, cervical cancer screening, electrocardiogram, and health checkup informed on site, and then performed a cross-sectional study to analyze the distribution and significance of critical values of 4,721 participants from different views and the association relation of 628 participants with more than one critical value and a retrospective cohort study to analyze the incidence and dynamic evolution of critical values based on 2,813 participants attending the physical examination from 2017 to 2019.

**Results:** A total of 4,721 participants were included in the retrospective study. The prevalence of 10 critical values from 33,639 participants was over 0.6%. The critical values of obesity, hypertension, Glucose_T, Liver_T, Kidney_T, Lipid_T, Urine_T, and Head_CT were significantly increased in men (*P* < 0.05), whereas the results were the opposite for the Blood_T and Thyroid_US (*P* < 0.01). The prevalence trend of critical values increased along with age, where the prevalence of men was higher than that of women under 60 years old (*P* < 0.01), while the prevalence of women increased by four times and exceeded the prevalence of men above 70 years old. Association relation analysis identified 16 and 6 effective rules for men and women, respectively, where the critical values of Urine_T and Glucose_T played the central roles. Furthermore, a retrospective dynamic evolution analysis found that the incidence of new critical values was about 10%, the incidence of persistent critical values was about 50%, and that most of the effective evolution paths tended to no critical values for men and women.

**Conclusion:** In conclusion, this study provides a new perspective to explore the population health status using the critical value reporting data in a physical examination center, which can assist in decision-making by health management at the population level and in the prevention and treatment of various types of cancer and chronic diseases at the individual level.

## Introduction

In clinical activity, the term “critical value” was defined first by George D. Lundberg in 1972 as a laboratory test result that represents a pathophysiologic state at such variance with normal as to be life-threatening unless something is done promptly and for which some corrective action could be taken (1–4) and then plays an important role in ensuring patient safety and supporting effective clinical decision-making (5). Similarly, in health management and examination activity, there exist critical values in health checkup, which is represented as the important abnormal results of a certain system or an organ that is found by the physical examinee without symptoms and signs, including clinical critical values, major diseases and their clues, acute and chronic lesions, and abnormal examination results requiring dynamic observation (6). Thus, compared with clinical critical values, the critical value in health checkup has a much wider definition scope and a lower population ratio in the healthy population.

Recently, critical value research mainly involves two aspects: critical value reporting policies and practices and critical value data analysis. The critical value reporting is a key component to improve total healthcare system by encouraging health care providers for effective treatment of the patients; thus, many studies examine critical value reporting policies and practices and identify critical value ranges for different hospitals using a national survey (7–10). For the second aspect, many studies mainly analyze the occurrence and distribution of critical values and the relationship between the frequency of such values and patient outcome and can provide information for hospitals on improving reporting policies (11–15). In summary, most of these studies focus on laboratory critical values and also the lack of the association relation and dynamic evolution analysis over time for critical values.

In addition, few studies have formally analyzed and discussed the critical values in health checkup. In China, the malignant tumor has become one of the major public health problems. According to the latest statistics, the death rate of malignant tumor accounts for 23.91% of the total death causes of residents, and the incidence and the mortality have been increasing in the recent decade; thus, early prevention, early screening, early diagnosis, and early treatment of malignant tumors are very important. Critical values in health checkup are the early symptoms and an inevitable process of malignant tumors, and the analysis of critical values is a crucial task in a health management center (16). Hence, in this study, our objectives are to (1) examine the prevalence of the critical values in health checkup at the healthy population level to find the possible relationship with the incidence of malignant tumors, (2) explore the association relation among the critical values to find the core critical values, and (3) carry out the retrospective cohort analysis to acquire the incidence of new and persistent critical values and a dynamic evolution trend of critical values in health checkup over time.

## Methods

### Participant Information

**Figure 1:**
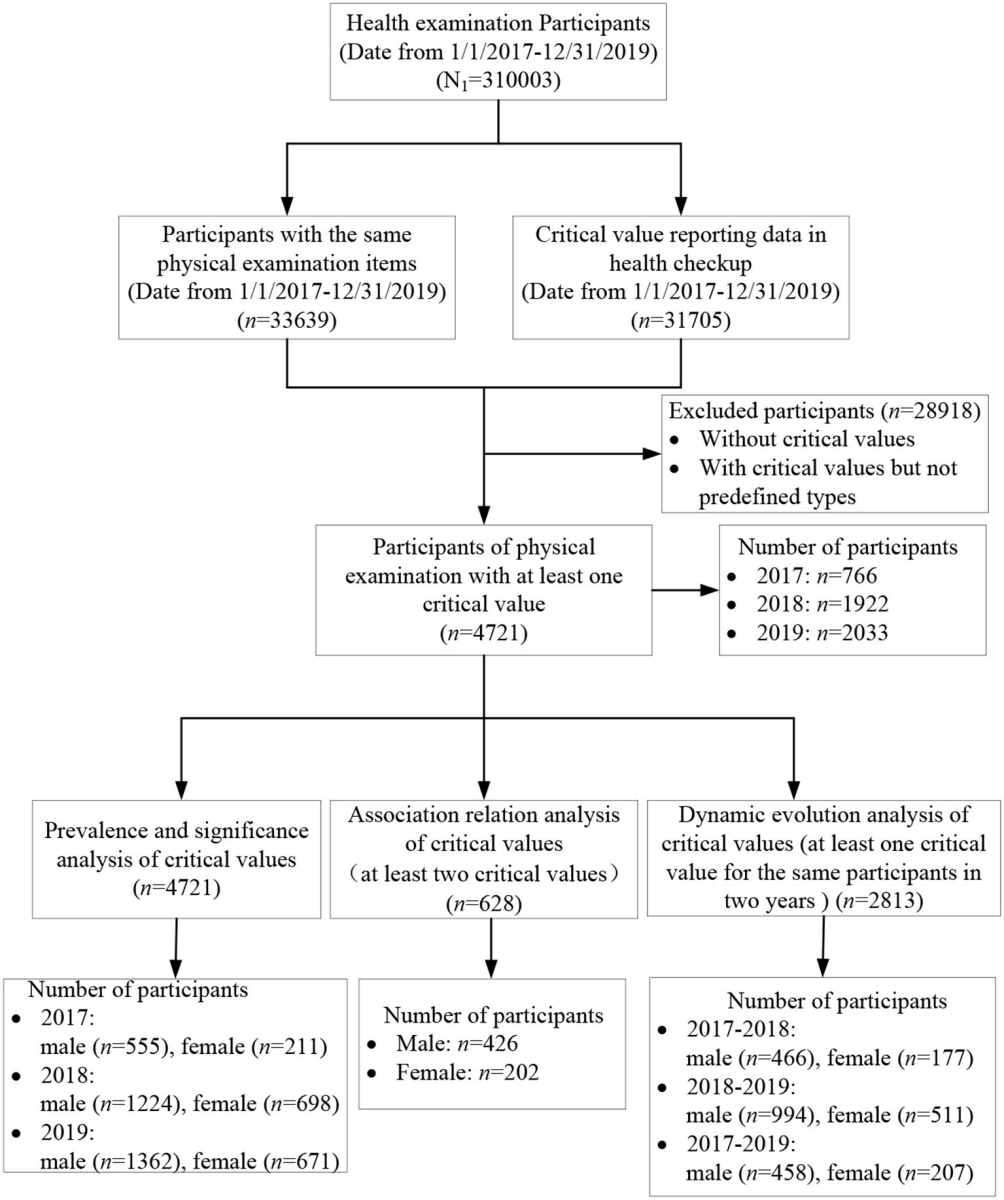
Study design and flow diagram.

This study was conducted in accordance with the Helsinki Declaration and Rules of Good Clinical Practice. The study was approved by the Institutional Review Board of the First Affiliated Hospital of Zhengzhou University (2018-KY-56). From January 2017 to December 2019, there were a total of 33,639 physical examiners with the same physical examination items from the Health Management Center, the First Affiliated Hospital of Zhengzhou University in Central China. The same items are the basis of the critical value analysis in health checkup because it is difficult to make sure whether the participants have this critical value if they do not take this examination. The physical examination items mainly include a laboratory test (blood routine test, urine routine test, liver function, renal function, blood lipid, and blood glucose) by a Roche cobas 8000 automatic biochemical analyzer, ultrasound examination items (breast, thyroid, abdominal ultrasonography, etc.) by a Philips Affiniti 50 color Doppler ultrasound system with a linear array probe and a frequency of 5–12 MHz, cervical cancer screening items [Thinprep Cytologic Test (TCT) by cytological diagnosis based on Bethesda systematic classification, Human Papillomavirus (HPV) by a Roche cobas HPV detection kit], electrocardiogram by Electrocardiograph (MedEx, MECG-300, China), CT by a spectral CT scanner (Discovery CT, GE Healthcare), height and weight measurement for body mass index (BMI) by an ultrasonic height and weight meter with SK-X80 (SONKA, China), and blood pressure measurement by a medical electronic sphygmomanometer (OMRON, China). The same physical examination items used the same devices and equipment from 2017 to 2019. After removing 28,918 participants without critical values, the remaining 4,721 participants were used for statistical analysis, including the prevalence and significance analysis, association relation, and dynamic evolution analysis (Figure 1).

### Definition of the Critical Values in Health Checkup

According to the expert consensus on the management of important results with abnormal values in physical examination, the clinical experts from the health management center and relevant departments jointly study and determine the critical value lists and submit them to the medical quality committee of the hospital for approval. The critical value list includes two categories: one is highly abnormal and endangering the life safety of the physical examinees and the other is some important positive findings that may affect the life and health of the physical examinees, although they will not endanger the life and health in a short time. The critical value lists include the test items, analytes, and ranges (Tables 1–4).

**Table 1:** The critical value list in health checkup for a laboratory test.

**Test items**	**Analytes**	**Critical value range**
Routine Blood Test (Blood_T)	WBC count	≤ 2 × 10^9^/L, ≥20 × 10^9^/L
	Platelet count	≤ 30 × 10^9^/L, ≥1000 × 10^9^/L
	Hemoglobin	≤ 60 g/L
Blood Glucose Test (Glucose_T)	Glucose	≥16.7 mmol/L and ≥13.9 mmol/L with strong positive urine ketone body
	Glycosylated hemoglobin	≥15%
Liver Function Test (Liver_T)	Alanine aminotransferase	≥100 U/L
	Aspartate aminotransferase	≥100 U/L
	Glutamyl transpeptidase	≥200 U/L
	Total bilirubin	≥85.5 μmol/L
Kidney Function Test (Kindney_T)	Creatinine	>115 μmol/L
Blood Lipid Test (Lipid_T)	Triglyceride	≥15 mmol/L
Thyroid Function Test (Thyroid_T)	Free triiodothyronine	≥2 × maximum reference value
	Free thyroxine	
	Thyroid stimulating hormone	
Routine Urine Test (Urine_T)	Occult blood	≥3 +
	Pathological cast	≥1/μL
	Transparent tube type	≥2/μL
	Ketone body	≥2 +
	Glucose	≥4 +
	Protein	≥3 +
Tumor Marker Test (Tumor_T)	Alpha fetoprotein, Carcinoembryonic antigen	≥2 × maximum reference value
	Ca72-4, Ferritin	≥3 × maximum reference value
	Ca125, Ca19-9	≥maximum reference value
	Tumor abnormal protein	≥160 μm^2^
	Total prostate specific antigen	≥4 ng/ml

**Table 2:** The critical value list in health checkup for imaging.

**Examination items**	**Critical value list**
Thyroid Ultrasound (Thyroid_US)	TI-RADS ≥ category 4; Parathyroid adenoma; Cervical lymph nodes enlargement; Considering lymph node metastasis.
Cardiac Ultrasound and Computed Tomography (Cardiac_US&CT)	Ventricular aneurysm; Severe reflux; Ascending aorta widening (widening > 45 mm); Pericardial effusion (large amount); Moderate-severe stenosis (moderate-severe incomplete closure); Aortic aneurysm; Coronary artery stenosis degree > 75%.
Abdominal Ultrasound (Abdominal_US)	Liver cirrhosis; Ascites; Severe fatty liver; Liver cyst; Solid space occupying lesions (retroperitoneal space occupying lesions); Solid nodules (further examination); Intrarenal hyperechogenicity (further examination); Intrahepatic and extrahepatic bile ducts Widening (or high limit); Pancreatic lesions (any lesions); Separation of collecting system; Massive pleural effusion; Intrahepatic low-density shadow.
Neck Vascular Ultrasound (Neck_US)	Carotid artery stenosis > 50; Moderate-severe stenosis; Internal carotid artery occlusion; Left cervical lymph node enlargement.
Urinary System Ultrasound (Urinary_US)	Hydronephrosis (moderate-severe); Small renal volume; Localized thickening of bladder wall; Ureteral dilatation; Separation of renal collecting system; Prostate.
Pelvic Ultrasound (Pelvic_US)	Ectopic pregnancy.
Breast Ultrasound and Mammography (Breast_US&MG)	BI-RADS category 0 and ≥ category 4A; Space occupying; Shadow.
Lung Computed Tomography and Chest X-Ray (Lung_CT&X)	Space occupying; Mediastinum widened; Hilar widened (slightly larger plus data); Pleural effusion (medium and large amount); Suspicious small nodules; Abnormal density shadow; Ground glass nodules.
Head Computed Tomography (Head_CT)	High pathological changes >80%; Cerebral vascular stenosis; Cerebral hemorrhage; Aneurysms; Subacute cerebral infarction; Arachnoid cyst; Cerebral hemorrhage; New cerebral infarction; Brain abscess and other signs of canceration.

**Table 3:** The critical value list in health checkup for cervical cancer screening and electrocardiogram.

**Test or Examination items**	**Critical value list**
Thinprep Cytologic Test and Human Papilloma Virus (TCT&HPV)	TCT: Non benign/reactive lesions; Malignant cells; Low-grade and high-grade intraepithelial lesions; Atypical squamous cells; HPV-16/18 positive.
Electrocardiogram(ECG)	Ventricular premature beats (biphasic, triplet, quadruple, multifocal, frequent, ventricular tachycardia, arrest); Atrioventricular block, ventricular arrest and occasional sinus arrest were more than type 2; High potassium, low potassium, low blood calcium, visible atrial escape rate; High and sharpT wave, ST segment elevation (arch back upward) or significantly depressed with T wave inversion; Sinus bradycardia <45, accompanied by long interval of 3S, QT prolongation; Acute myocardial infarction/Brugada wave.

**Table 4:** The critical value list in health checkup informed on site.

**Measurements items**	**Critical value list**
Measurement of height and weight(Obesity)	Body mass index (BMI) = weight/(height)^2^: BMI≥30(Kg/m^2^)
Blood pressure measurement(Hypertension)	Systolic blood pressure (SBP)≥180(mmHg) or Diastolic blood pressure (DBP)≥110(mmHg)

### An Effective Association Rule and Dynamic Evolution Path Identification

In this study, an association rule (X ≥ Y) refers to the occurrence of a critical value (Y) based on another critical value X, while an effective and strong association rule must meet three conditions, including the support, confidence, and lift. The support [*p* (X, Y)] is the co-occurrence frequency of the critical values X and Y, the confidence [*p* (Y/X)] measures the possibility that the critical value Y occurs after suffering from the critical value X, and the lift [*p* (Y/X)/*p*(Y)] measures the promotion function of critical value Y after the critical value X, reflecting the effectiveness of relation between the critical value X and the critical value Y. Hence, an effective association rule can be identified when the support and the confidence exceed the minimum values defined beforehand, and also the lift is >1.

A dynamic evolution path (Y → Z) refers to the transition of the critical value Z after the critical value Y in two consecutive years, reflecting the persistence and variability of the critical values. Thus, an effective evolution path can be identified when the transition probability of the critical value Y in 1 year and Z in the next year exceeds the minimum values defined beforehand.

### Statistical Analysis

Statistical analysis were performed using R software. R software was used to analyze the prevalence and significance, construct the association rule model (R 4.0.2; an arules and arulesViz package), and identify the dynamic evolution path of critical values in health checkups from 2017 to 2019. A cross-sectional study was performed for the single participant to determine the prevalence and association among critical values in health checkups. A retrospective cohort study was performed for the incidence of emerging and persistent critical values for the participants with follow-up physical examination. The age value is presented as the mean ± SD. The Student's *t*-test was used to evaluate the differences between the sets of continuous variables, the chi-square test was used to evaluate the differences between the sets of categorical variables, and there were significant differences between observation groups with and without critical values in health checkup when *P* is < 0.05.

## Results

### Participant Demographics

A total of 33,639 participants (19,185 males and 14,454 females) were extracted in this study from January 2017 to December 2019. Then, 28,918 participants without critical values were excluded, and the remaining 4,721 participants were identified for further data analysis. Among 4,721 participants, 4,093 participants have only one critical value, 539 participants have two critical values, 72 participants have three critical values, 15 participants have four critical values, and 2 participants have five critical values. All the 4,721 participants are affiliated with 107 units, such as universities, banks, enterprises, and institutions, as well as other social organizations. The mean age was 49.32 ± 13.87 years old, where the youngest and the oldest were 21 and 96 years old, respectively. Specifically, the numbers of male and female participants were 3,141 and 1,580, respectively, the mean ages were 48.50 ± 13.50 and 50.95 ± 14.43 years old, respectively, and there was a significant difference in years (*P* < 0.05). In addition, the number of participants gradually increased from 766 in 2017 and 1,922 in 2018 to 2,033 in 2019, since the total amount of physical examinees in 2017 is far less than that in 2018 and 2019.

**Figure 2:**
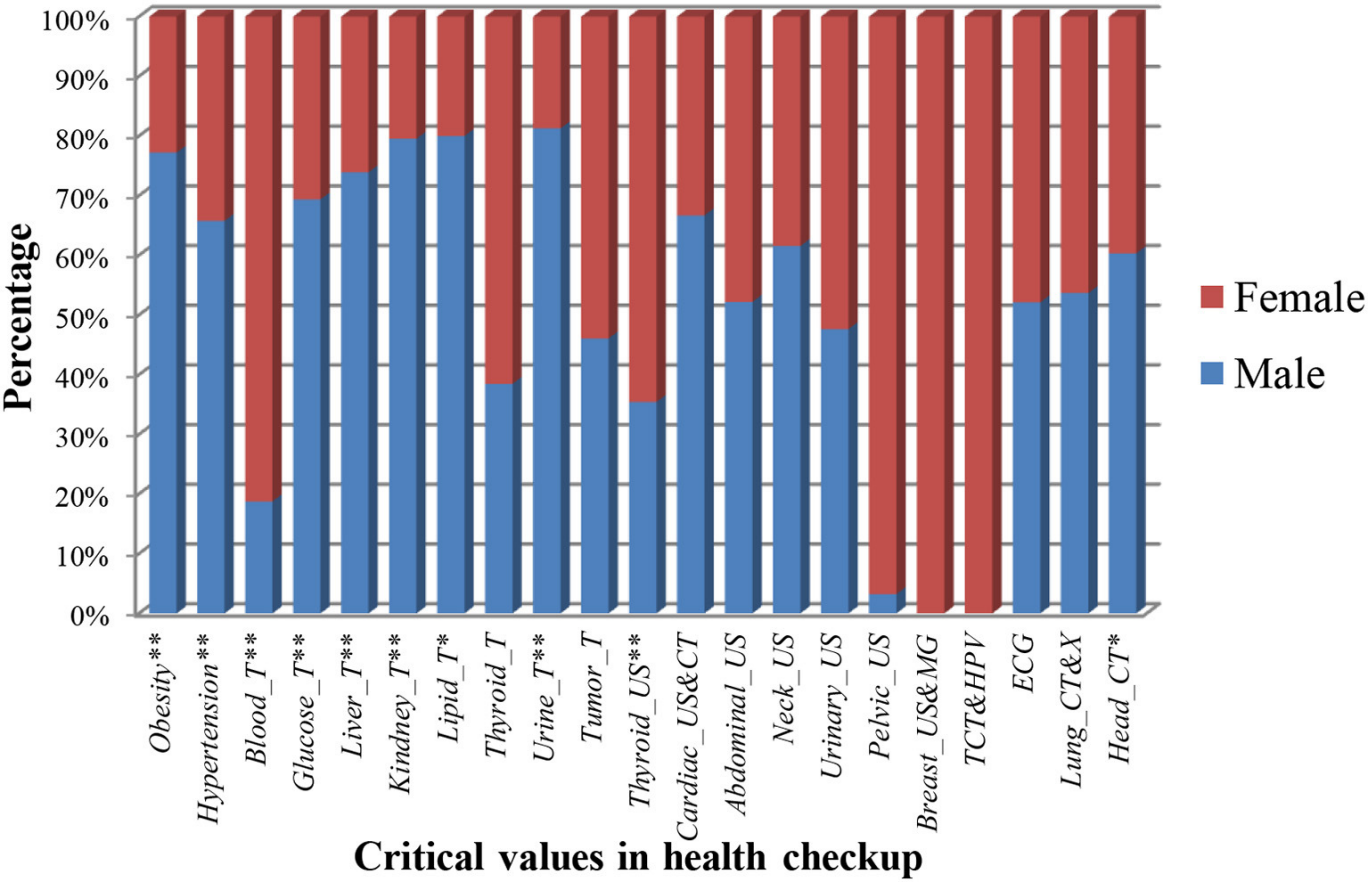
The prevalence of critical values in health checkup for different genders. * and ** denote the statistically significant difference in the 0.05 and 0.01 tested levels.

After analyzing the critical value data of 4,721 participants, the prevalence and significance of critical values in health checkups were shown in Table 5 and Figure 2. In summary, the prevalence of critical values was 14.03, 16.37% for men and 10.93% for women. Among the prevalence of all the critical values over 0.6%, obesity accounted for the highest percentage with the prevalence of 6.40% for all the participants, 9.18% for men and 2.71% for women, followed by thyroid_US (1.69%), hypertension (1.54%), ECG (0.95%), lung_CT and X (0.83%), tumor_T (0.81%), liver_T (0.74%), TCT and HPV (0.67%), Breast_US and MG (0.64%), and urine_T (0.61%) for all the participants; hypertension (1.94%), thyroid_US (1.25%), liver_T (1.02%), ECG (0.99%), urine_T (0.91%), lung_CT and X (0.88%), and liver_T (0.74%) for male participants; thyroid_US (2.28%), hypertension (1.01%), ECG (0.91%), tumor_T (0.89%), lung_CT and X (0.76%), TCT and HPV (0.67%), and breast_US and MG (0.64%) for female participants.

**Table 5:** The prevalence and significance analysis of critical values in health checkup.

**Critical value**	**Total (*N* = 33,639)**	**Male (*n* = 19,185)**	**Female (*n* = 14,454)**	***x*^**2**^**	***P*-Value**
Obesity	2154 (6.40%)	1762 (9.18%)	392 (2.71%)	576.17	<0.01^**^
Hypertension	519 (1.54%)	373 (1.94%)	146 (1.01%)	47.35	<0.01^**^
Blood_T	75 (0.22%)	18 (0.09%)	57 (0.39%)	33.47	<0.01^**^
Glucose_T	86 (0.26%)	65 (0.34%)	21 (0.15%)	12.11	<0.01^**^
Liver_T	248 (0.74%)	196 (1.02%)	52 (0.36%)	49.35	<0.01^**^
Kindney_T	81 (0.24%)	68 (0.35%)	13 (0.09%)	24.01	<0.01^**^
Lipid_T	18 (0.05%)	15 (0.08%)	3 (0.02%)	5.08	0.024^*^
Thyroid_T	20 (0.06%)	9 (0.05%)	11 (0.08%)	1.18	0.277
Urine_T	205 (0.61%)	174 (0.91%)	31 (0.21%)	65.26	<0.01^**^
Tumor_T	274 (0.81%)	146 (0.76%)	128 (0.89%)	1.58	0.208
Thyroid_US	570 (1.69%)	240 (1.25%)	330 (2.28%)	52.72	<0.01^**^
Cardiac_US&CT	31 (0.09%)	23 (0.12%)	8 (0.06%)	3.73	0.053
Abdominal_US	117 (0.35%)	70 (0.36%)	47 (0.33%)	0.38	0.54
Neck_US	44 (0.13%)	30 (0.16%)	14 (0.1%)	2.24	0.135
Urinary_US	71 (0.21%)	39 (0.2%)	32 (0.22%)	0.13	0.72
Pelvic_US^†^	46 (0.3%)	2 (0.01%)	44 (0.3%)		
Breast_US&MG^†^	93 (0.64%)		93 (0.64%)		
TCT&HPV^†^	97 (0.67%)		97 (0.67%)		
ECG	321 (0.95%)	189 (0.99%)	132 (0.91%)	0.45	0.502
Lung_CT&X	278 (0.83%)	168 (0.88%)	110 (0.76%)	1.32	0.25
Head_CT	109 (0.32%)	73 (0.38%)	36 (0.25%)	4.41	0.036^*^
All	4721 (14.03%)	3141 (16.37%)	1580 (10.93%)	206.16	<0.01^**^

* *Denotes a statistically significant difference (P < 0.05)*.

** *Denotes a statistically significant difference (P < 0.01)*.

† *Only for the female*.

Moreover, in Table 5 and Figure 2, the total number and prevalence of critical values in men were higher than that in women (*P* < 0.01), hinting that men were prone to present abnormal items. More specifically, compared with women, the prevalence of obesity, hypertension, glucose_T, liver_T, kidney_T, lipid_T, urine_T, and head_CT were significantly increased in the male population (*P* < 0.05), whereas for blood_T and thyroid_US, the female population presented an increased percentage compared with the male population (*P* < 0.01). In addition, the prevalence of tumor_T, cardiac_US and CT, abdominal_US, neck_US, urinary_US, ECG, and lung_CT and X in men and women had no significant difference, indicating that both suffered from these abnormal items with the same risk. Lastly, pelvic_US, BCDU and M, and TCT and HPV only existed in women.

**Figure 3:**
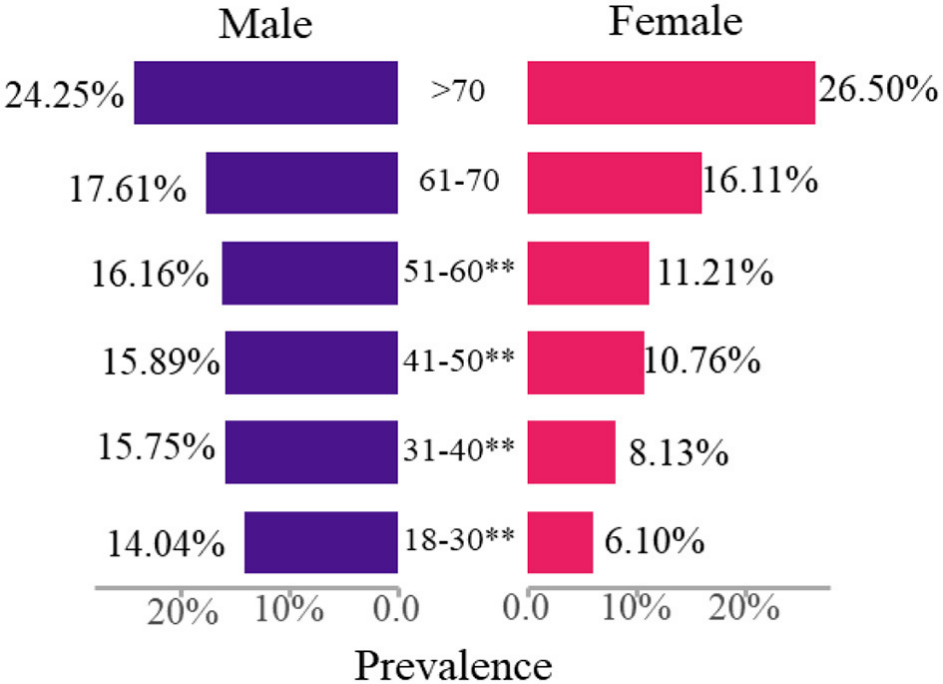
The prevalence trend of critical values along with age for the male and female. **Denotes a statistically significant difference (*P* < 0.01).

Furthermore, we analyzed the difference analysis of critical values for different genders and ages, acquired the results of the top six with the highest prevalence (Table 6), and the prevalence trend of critical values along with age (Figure 3). The prevalence of obesity and hypertension in men was higher than that in women under 70 years old, while the opposite was true for thyroid_US. The prevalence of urine_T, ECG, and lung_CT and X had no significant difference in men and women. More importantly, the prevalence of all critical values increased to about 25% along with age, where the prevalence of men was higher than that of women under 60 years old (*P* < 0.01), while the prevalence of women increased by four times and exceeded the prevalence of men above 70 years old.

**Table 6:** The significance analysis of critical values for different ages and genders.

**Age group**		**Obesity**	**Hypertension**	**Thyroid _US**	**Urine _T**	**ECG**	**Lung _CT&X**	**Total**
18–30	Male (1,090)	106	2	8	11	6	5	153 (14.04%)
	Female (951)	12	1	17	15	8	8	58 (6.10%)
	*X*^2^	66.53		4.66	1.27	0.63	1.17	34.52
	*P*-Value	<0.01^**^		0.03^*^	0.25	0.43	0.279	<0.01^**^
31–40	Male (5,676)	640	58	53	38	28	23	894 (15.75%)
	Female (4,673)	103	9	93	43	28	27	380 (8.13%)
	*X*^2^	316.49	27.40	20.56	2.07	0.53	1.59	137.81
	*P*-Value	<0.01^**^	<0.01^**^	<0.01^**^	0.15	0.47	0.21	<0.01^**^
41–50	Male (5,331)	505	126	63	45	41	43	847 (15.89%)
	Female (3,811)	82	23	93	43	28	27	410 (10.76%)
	*X*^2^	198.24	42.94	20.99	1.88	0.04	0.28	149.31
	*P*-Value	<0.01^**^	<0.01^**^	<0.01^**^	0.17	0.85	0.6	<0.01^**^
51–60	Male (4,432)	327	81	72	32	59	46	716 (16.16%)
	Female (2,945)	76	26	85	14	26	18	330 (11.21%)
	*X*^2^	78.85	11.05	13.52	1.74	3.12	3.75	35.62
	*P*-Value	<0.01^**^	<0.01^**^	<0.01^**^	0.19	0.08	0.05	<0.01^**^
61–70	Male (1,681)	116	59	24	14	25	26	296 (17.61%)
	Female (1397)	66	29	46	8	18	24	225 (16.11%)
	*X*^2^	6.50	5.65	11.94	0.73	0.22	0.14	1.23
	*P*-Value	0.011^*^	0.017^*^	<0.01^**^	0.39	0.64	0.71	0.268
>70	Male (969)	68	47	20	6	30	25	235 (24.25%)
	Female (668)	53	58	14	0	23	16	177 (26.50%)
	*X*^2^	0.485	9.67	0		0.15	0.06	1.06
	*P*-Value	0.486	<0.01^**^	0.97		0.70	0.81	0.304

* *Denotes a statistically significant difference (P < 0.05)*.

** *Denotes a statistically significant difference (P < 0.01)*.

### Association Relation Analysis

**Figure 4:**
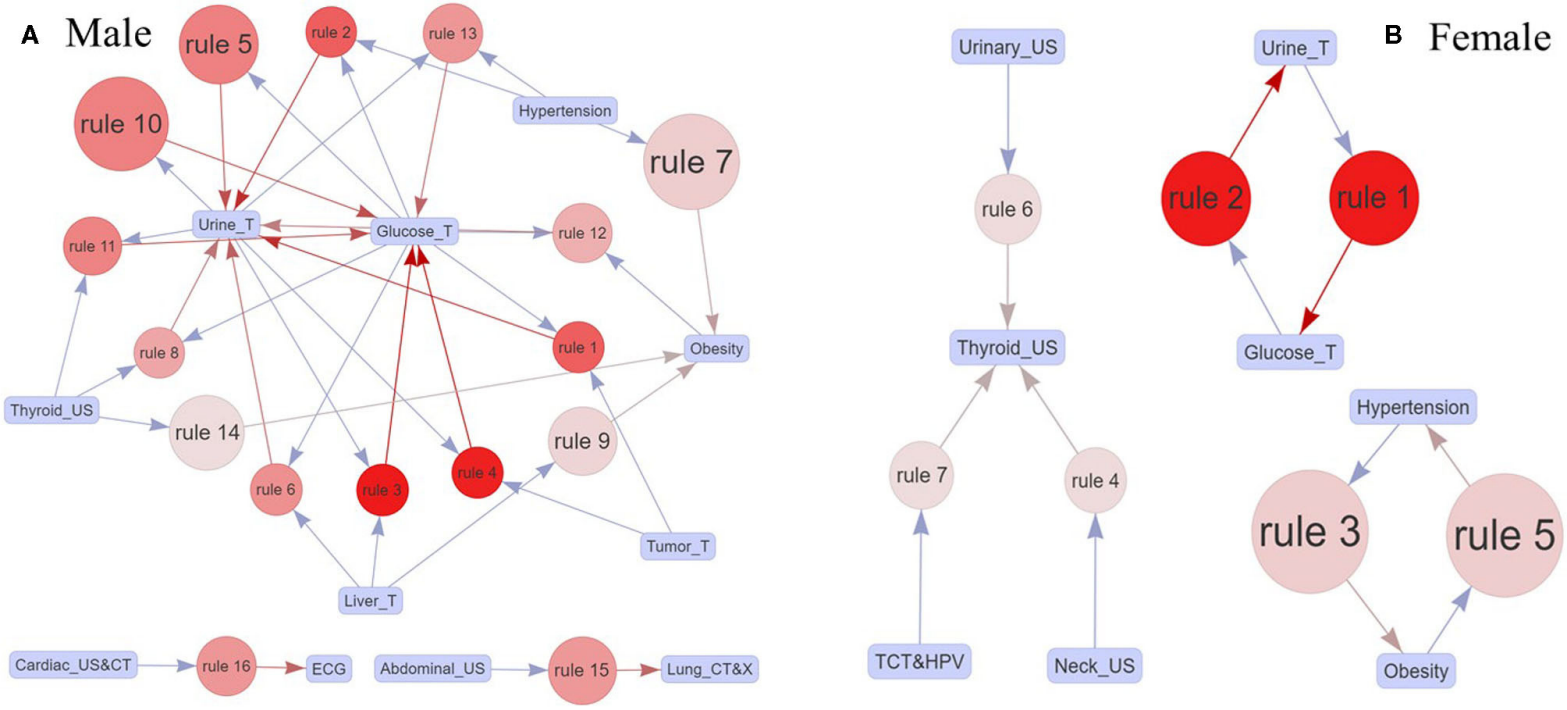
**(A,B)** The effective association rules for the male and the female participants. A rule was composed of boxes, arrows, and a circle; the blue and red arrows were the critical values on LHS and RHS; and the size and the color of the circle showed the support of the critical value on LHS and the lift of the rule, respectively.

A total of 629 participants with more than one critical value were selected to analyze the association relation, including 426 males and 203 females. After adopting the Apriori algorithm (17) and defining the minimum support, confidence, and lift as 0.01, 0.4, and 1, we finally identified 16 and seven effective rules for the male and the female participants (Table 7, Figure 4). An effective rule can be represented by a group of critical values from the left hand side (LHS) to the right hand side (RHS).

**Table 7:** The detailed results of the identified effective and strong rules.

**Gender**	**No**.	**Rule (LHS ≥ RHS)**	**Support**	**Confidence**	**Lift**	**Count**
Male	1	{Glucose_T,Tumor_T} ≥ {Urine_T}	0.01	1	4.44	6
	2	{Glucose_T,Hypertension} ≥ {Urine_T}	0.01	1	4.44	5
	3	{Liver_T,Urine_T} ≥ {Glucose_T}	0.02	0.88	5.82	7
	4	{Tumor_T,Urine_T} ≥ {Glucose_T}	0.01	0.86	5.71	6
	5	{Glucose_T} ≥ {Urine_T}	0.13	0.84	3.74	54
	6	{Glucose_T,Liver_T} ≥ {Urine_T}	0.02	0.78	3.45	7
	7	{Hypertension} ≥ {Obesity}	0.19	0.71	1.34	83
	8	{Glucose_T,Thyroid_US} ≥ {Urine_T}	0.01	0.63	2.77	5
	9	{Liver_T} ≥ {Obesity}	0.08	0.56	1.07	36
	10	{Urine_T} ≥ {Glucose_T}	0.13	0.56	3.74	54
	11	{Thyroid_US,Urine_T} ≥ {Glucose_T}	0.01	0.56	3.7	5
	12	{Glucose_T,Obesity} ≥ {Urine_T}	0.01	0.56	2.47	5
	13	{Hypertension,Urine_T} ≥ {Glucose_T}	0.01	0.5	3.33	5
	14	{Thyroid_US} ≥ {Obesity}	0.06	0.44	0.82	27
	15	{Abdominal_US} ≥ {Lung_CT&X}	0.04	0.43	3.23	17
	16	{Cardiac_US&CT} ≥ {ECG}	0.01	0.42	3.35	5
Female	1	{Urine_T} ≥ {Glucose_T}	0.07	0.75	7.58	15
	2	{Glucose_T} ≥ {Urine_T}	0.07	0.75	7.58	15
	3	{Hypertension} ≥ {Obesity}	0.13	0.6	1.92	27
	4	{Neck_US} ≥ {Thyroid_US}	0.01	0.43	1.31	3
	5	{Obesity} ≥ {Hypertension}	0.13	0.43	1.92	27
	6	{Urinary_US} ≥ {Thyroid_US}	0.02	0.42	1.28	5

Specifically, men identified 16 rules with 11 critical values, and the core critical values were urine_T and glucose_T. The rule {hypertension} ≥ {obesity} (rule 7) had the maximum support with 0.19, indicating that the combination of hypertension and obesity was particularly prevalent in men. Then, the rules with the second largest support were {glucose_T} ≥ {urine_T} (rule 5) and {urine_T} ≥ {glucose_T} (rule 10), where the confidence of the former is larger than that of the latter. Also, the confidence values of the first two rules ({glucose_T, tumor_T} ≥ {urine_T}, {glucose_T, hypertension} ≥ {urine_T}) were 1, showing the critical values on the RHS appeared certainly along with the critical values on the LHS. While for women, there were only six rules with seven critical values and three decentralized association networks, including urine_T, glucose_T, hypertension, obesity, thyroid_US, urinary_US, and neck_US. Similarly, the combination of hypertension and obesity was prevalent in women, since rules 1 and 5 had the maximum support. Meanwhile, the rules {urine_T} ≥ {glucose_T} (rule 1) and {glucose_T} ≥ {urine_T} (rule 2) had the same support, confidence, and lift.

### Dynamic Evolution Analysis

**Figure 5:**
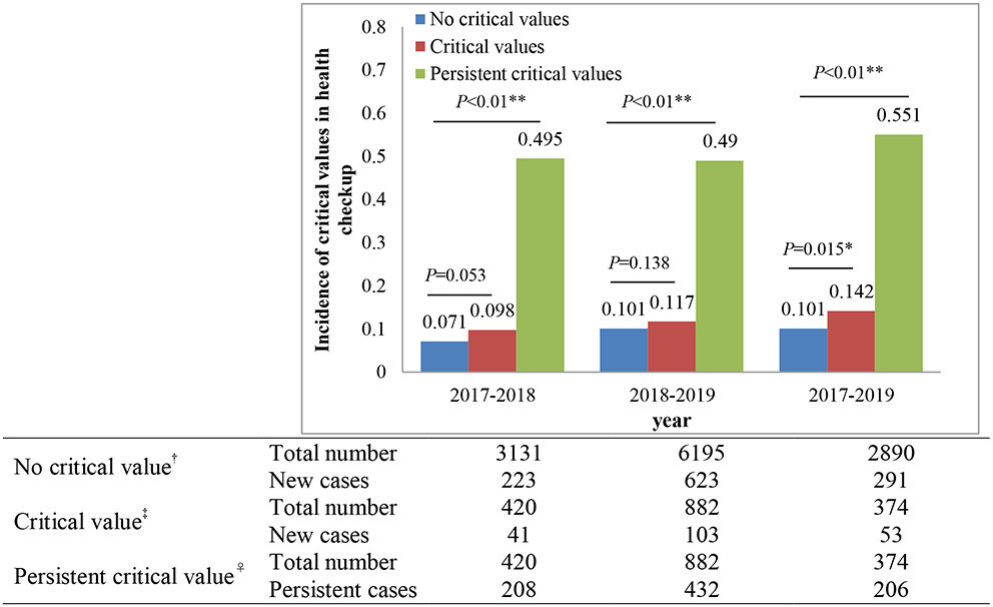
The incidence of emerging and persistent critical values for the participants with follow-up physical examination. ^†^Denotes no critical value in the previous year but new critical values in the next year, ^‡^denotes critical values in the previous year and new critical values in the next year, and ^♀^denotes critical values in the previous year and the next year. *Denotes a statistically significant difference (*P* < 0.05), **denotes a statistically significant difference (*P* < 0.01).

**Figure 6:**
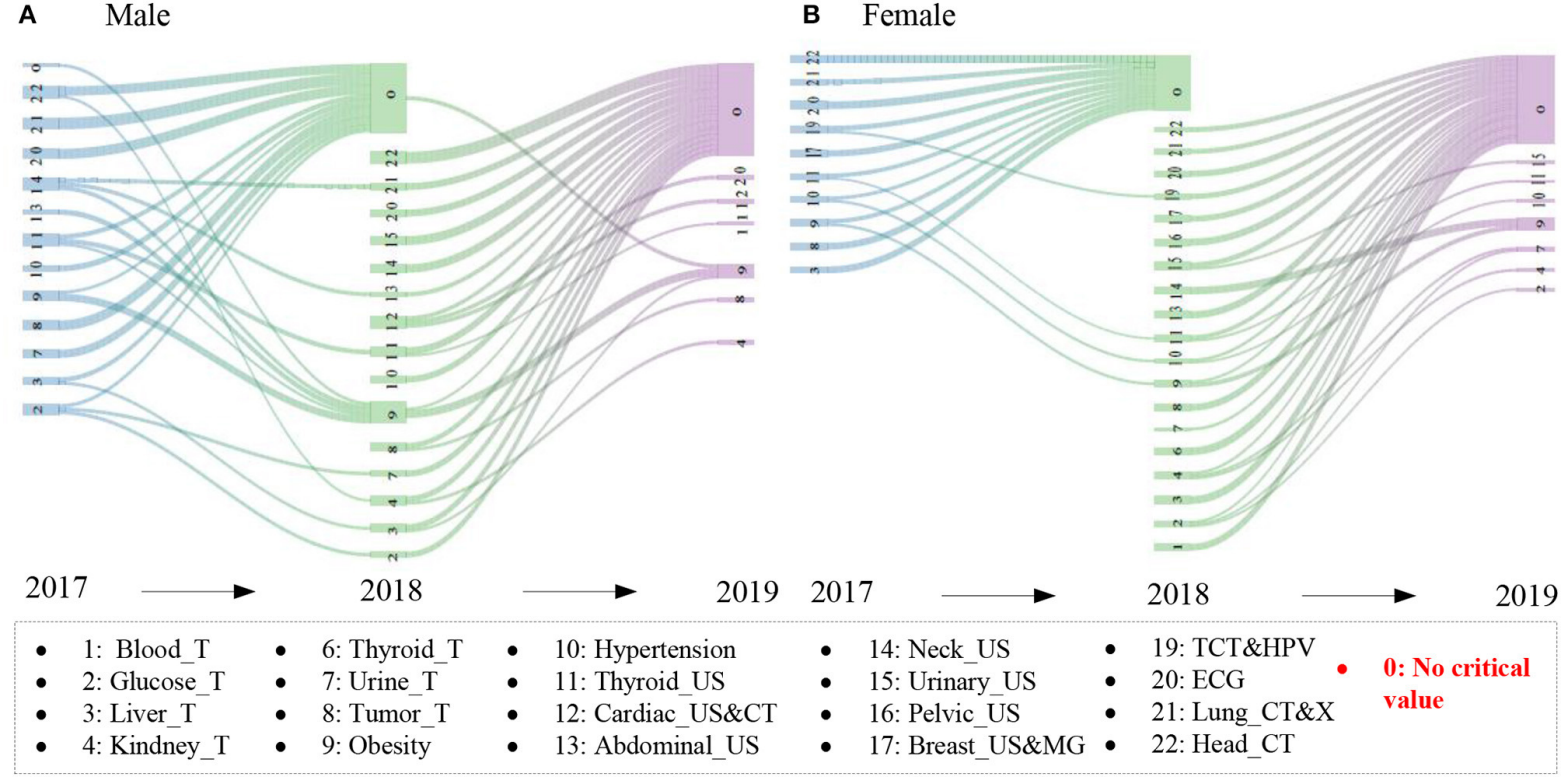
The effective evolution paths for men and women. 2017 → 2018 and 2018 → 2019 denote the same participants with at least one critical value in 2017 and 2018 and 2018 and 2019, respectively. The critical values were shown when the transition probability exceeded 0.2.

A total of 2,813 participants with more than one critical value attending the physical examination in 2 years from 2017 to 2019 were selected to analyze the dynamic evolution, including 643 participants (466 males and 177 females) in 2017 and 2018, 1,505 participants (994 males and 511 females) in 2018 and 2019, and 665 participants (458 males and 207 females) in 2017 and 2019. Figure 5 shows the incidence of emerging and persistent critical values for the participants with follow-up physical examination, and Figure 6 shows the effective evolution paths for men and women from 2017 to 2019, after defining the minimum transition probability as 0.2.

In Figure 5, we found that the incidence of new critical values was from 7 to 14%, whereas the incidence of persistent critical values was about 50%. In addition, whether there was a critical value in 2017 and 2018 or not, the incidence of new critical values in 2018 and 2019 had no significant difference (*P* = 0.053, *P* = 0.183). Interestingly, if there exist critical values in 2017, the incidence of new critical values in 2019 was significantly higher than that without critical values in 2017 (*P* = 0.015). Also, the incidence of persistent critical values had a significantly higher than the incidence of new critical values in 2017 → 2018 (*P* < 0.01), 2018 → 2019 (*P* < 0.01), and 2017 → 2019 (*P* < 0.01).

In Figure 6, 20 and 23 effective evolution paths were identified in 2017 → 2018 and 2018 → 2019 for men, while 15 and 28 effective evolution paths were identified in 2017 → 2018 and 2018 → 2019 for the women, respectively. An effective evolution path can be represented by a critical value in 1 year to a critical value in the next year, that is, A → B, which reflects the persistence and variability of critical values. The path of critical values → 0 was the most popular, indicating the participants paid attention to the critical value and timely prevented, intervened, and treated these diseases after the physical examination; thus, no critical values were found in the next year. In addition, there existed larger persistence for many critical values, especially obesity and thyroid_US. Also, the variability among critical values in different years existed, such as abdominal_US → obesity, hinting at the mutual influence relationship among diseases.

## Discussion

Health management is a process of comprehensive management of individual or population health risk factors. Its characteristics determine that the critical value in health checkup covers a wider range than that of the clinical critical value. We first defined the critical value list in health checkup according to the clinical practice, including 19 abnormal items and the corresponding critical value types and the range, such as for the WBC count and the platelet count in the routine blood test, the clinical critical value ranges were “ ≤ 1.5 × 10^9^/L” and “≥50 × 10^9^/L,” “ ≤ 20 (a non-hematological ward) or 10 (a hematological ward)” and “≥1,000 × 10^9^/L” (14), while the critical values in health checkup were “ ≤ 2 × 10^9^/L” and “≥20 × 10^9^/L,” “ ≤ 30 × 10^9^/L” and “≥1,000 × 10^9^/L,” respectively. Moreover, for radiology and ultrasound examination, the number of clinical critical values was far less than that of the critical value in health checkup (12, 18).

In health examination report interpretation, critical value report, and follow-up practice, the clinician usually treated the critical value in health checkup as the high-risk factors of serious diseases to develop health intervention regimens. Also, critical values are the early symptoms of various types of cancer and the most direct reflection of the non-infectious chronic diseases (NCDs). Our results identified 10 critical values over.6% in 33,639 participants, that is, obesity (6.4%), thyroid_US (1.69%), hypertension (1.54%), ECG (0.95%), lung_CT and X (0.83%), tumor_T (0.81%), liver_T (0.74%), TCT and HPV (0.67%), breast_US and MG (0.64%), and urine_T (0.61%) for all the participants. The prevalence of many critical values in men was significantly higher than that in women, such as obesity, hypertension, glucose_T, liver_T, kidney_T, lipid_T, urine_T, and head_CT, while blood_T and thyroid_US are the opposite. Furthermore, for different age groups, men under 70 years old have a higher prevalence of obesity and hypertension than that of women, while thyroid_US is the opposite. Interestingly, the prevalence of the total critical values increased to about 25% along with age, which was always higher in men than in women under 60 years old (*P* < 0.01), while the opposite is true for those above 70 years old. According to the new report of cancer epidemiology in China (2015), there were about 3.929 million people who suffered from cancer and 2.338 million people died, and the incidence rate and the mortality rate of cancer continued to rise (19, 20). Also, NCDs, such as cardiovascular disease (CVD), diabetes, cancers, and osteoporosis, are the leading causes of death and health costs. According to the 2020 Report on Chinese Resident's Chronic Disease and Nutrition, NCDs account for 88.5% of the disease burden of China in 2019, more than all other causes combined (21). Thus, according to the relationship between NCDs and critical values, we summarized the incidence, prevalence, and mortality of malignant tumors, some common NCDs, and the corresponding critical values (Table 8).

**Table 8:** The incidence and the mortality of malignant tumors and NCDs with partial critical values.

**Top 10 malignant tumors**	**Incidence (1/100,000)**			**Mortality (1/100,000)**			**Critival values in health checkup**	**Prevalence (1/100,000)**		
	**All**	**Male**	**Female**	**All**	**Male**	**Female**		**All**	**Male**	**Female**
Lung	57.26	73.9	39.78	45.87	61.52	29.43	Lung_CT&X	830	880	760
Stomach	29.31	39.95	18.15	21.16	28.59	13.37				
Colorectum	28.2	31.96	24.25	13.61	15.56	11.58				
Liver	26.92	38.98	14.26	23.72	34.31	12.6	Liver_T; Abdominal_US	740; 350	1,020; 360	360; 330
Breast^†^	45.29		45.29	10.5		10.5	Breast_US&MG	640		640
Esophagus	17.87	25.13	10.25	13.68	19.45	7.62				
Thyroid	14.6		22.56				Thyroid_T; Thyroid_US	60; 1,690	50; 1,250	80; 2,280
Cervix	16.56		16.56			5.04	TCT&HPV; Pelvic_US	670; 300		670; 300
Brain,CNS	7.72	7.04	8.43	4.1	3.1	3.77	Head_CT	320	380	250
Pancreas	6.92	7.67		6.16	4.8	5.41	Abdominal_US	350	360	330
Prostate		10.23			4.36		Urinary_US	210	200	220
Bladder		8.83					Urinary_US	210	200	220
Lymphoma		7.43		3.62	4.38		Lung_CT&X	830	880	760
Uterus			10.28				Pelvic_US	300	10	300
Leukemia				3.96	3.2		Blood_T	220	90	390
Ovary						3.73				
Total	285.83	305.47	265.21	170.05	210.1	128	Tumor_T	810	760	890
**NCDs**	**Prevalence (1/100,000)**			**Mortality (1/100,000)**			**Critival values in health checkup**	**Prevalence (1/100,000)**		
	**All**	**Male**	**Female**	**All**	**Male**	**Female**		**All**	**Male**	**Female**
Hypertension and cardiovascular disease^‡^	25,200	26,200	24,100	271.8	296.4	264.4	Hypertension; Cardiac_US&CT; Head_CT	1,540; 90; 320	1,940; 120; 380	1,010; 60; 250
Diabetes^‡^	11,200						Glucose_T; Urine_T; Kindney_T	260; 610; 240	340; 910; 350	150; 210; 90
Dyslipidemia^‡^	40,400	47,000	33,500				Lipid_T	50	80	20
Total	23,000			533	611.2	452.6	Total^‡‡^	14,030	13,770	10,930

† *Only for the female*;

‡ *aged 18 and above in China*;

♀ *aged 40 and above in China*;

‡‡ *the total of critical values in health checkup*.

More concretely, lung cancer, upper digestive system tumor, liver cancer, colorectal cancer, and female breast cancer are still the main malignant tumors in China. The incidence and the mortality of lung cancer, stomach cancer, colorectum cancer, liver cancer, and esophagus in men are higher than that of in women, which is consistent with the prevalence of the critical values in Table 5 (male:female = 16.37:10.93%, *P* < 0.01). Furthermore, from the age distribution, the incidence of malignant tumors increases with age. It is below the level of young people under 40 years old, increases rapidly from 40 years old, mainly concentrates over 60 years old, and reaches the peak in the 80-year-old group (22). Similarly, our results also showed that the prevalence of critical values increased along with age and reached the maximum value above 70 years old, as shown in Table 6 and Figure 3. The incidence of thyroid cancer ranks fourth in women (22.56/100,000) and is significantly higher than that in men (23, 24). Also, our results found that the prevalence of thyroid_US in women (2.28%) was two times as many as that in men (1.25%) with a significant difference in the 0.01 tested levels in Table 5. Interestingly, the prevalence of thyroid_US in men was about 100 times more than the incidence of thyroid cancer in women, indicating that 1% of women with a critical value of thyroid_US (mainly TI-RADS 4A) was likely to develop thyroid cancer. Tumor markers are playing an increasingly important role in cancer screening and management, which can be specific for a certain type of cancer or may be present in different types of cancers (25). We found that the prevalence of abnormal tumor markers (810/100,000) was three times as much as the incidence of the total malignant tumors (285.83/100,000), hinting that one-third of abnormal tumor markers was likely to develop into malignant tumors. Thus, the critical values in health checkup could be regarded as an important warning of early malignant tumors (cancers).

In China, about 290 million people have suffered from CVD, and the main forms are hypertension, stroke, and coronary heart disease (26). According to the 2020 edition of the guideline of China for the prevention and treatment of type 2 diabetes, the prevalence of diabetes in China is increasing and now ranks first in the world, from 9.7 in 2010, 10.4 in 2013, and 11.2% in 2017 for people over 18 (27). However, our results showed that the prevalence of critical values is far lower than that of NCDs in Table 8, mainly including two reasons. One is the higher standard of critical values in health checkup than chronic diseases, such as hypertension III [SBP ≥ 180 (mmHg) or DBP ≥ 110 (mmHg)], indicating that the participants have suffered from very serious hypertension, and the blood pressure of many patients with hypertension cannot reach the defined critical value. The other reason is that many patients with chronic diseases have taken antihypertensive, hypoglycemic, and lipid-lowering drugs before the physical examination so that the measurement results cannot reach the defined critical value.

The results of association relation analysis showed that glucose_T and urine_T were the most typical abnormal items with the highest frequency for the male participants in Table 7 and Figure 4. More concretely, glucose_T and urine_T had a strong relation with most of the identified abnormal items, such as liver_T, lipid_T, thyroid_US, tumor_T, kidney_T, and ECG. First, for the abnormal glucose_T, the definition of critical values in health checkup indicated that the participants may have suffered from diabetes mellitus, where the glucose values were >16.7 mmol/L and 13.9 mmol/L with strong positive urine ketone body, or the glycosylated hemoglobin was >15% (28, 29). Diabetes mellitus was one of the most important public health challenges of the 21st century, which could lead to chronic damage, idiopathic pulmonary fibrosis, and dysfunction of various tissues, especially eyes, kidneys, heart, blood vessels, and nerves (30, 31). For the abnormal urine_T, the critical values in health checkup included the abnormal occult blood, pathological cast, transparent tube type, ketone body, glucose, and protein. Similarly, the urine_T is a basic indicator reflecting the health status of the body, which could directly and quickly reflect the situation of the urinary system and kidney metabolism and indirectly reflect some systemic diseases that affect the urine changes, such as diabetes mellitus, blood diseases, and hepatobiliary diseases (32, 33).

Furthermore, we found {hypertension} and {obesity} to be the most pervasive health problems in the Chinese population, since the rules had the highest support [male:{hypertension} ≥ {obesity}(rule 7, 19%), female:{hypertension} ≥ {obesity} and {obesity} ≥ {hypertension} (rules 3 and 5, 13%)]. The result of women participants showed that obesity also coexisted in 60% of the participants if they had suffered from hypertension, while the percentage was up to 71% in men. Also, the result of women participants showed the confidence of {hypertension} ≥ {obesity} (rule 3, 60%) was higher than {obesity} ≥ {hypertension} (rule 5, 43%), indicating that the emerging percentage of the obesity after hypertension exceeded that of hypertension after obesity. Thus, our results also indirectly proved the obesity is an important risk factor for hypertension (34). Besides, the results also identified two isolated rules in men, i.e., rule 15 ({abdominal_US} ≥ {lung_CT and X}) and rule 16 [(Cardiac_US&CT) ≥ (ECG)]. For rule 15, the reason may be that partial critical value of abdominal_US would be also found in the lung_CT and X, since the lower part of the lung is next to the liver, so, after scanning all the lungs by lung-CT, there must be “part” of the liver in the image (35). For rule 16, the abnormal cardiac_US and CT indicated that the participants had a high risk of heart diseases, for instance, coronary heart disease and cardiomyopathy, which are often accompanied by the abnormal ECG (e.g., ST segment elevation and Brugada wave) (36, 37). Hence, the abnormal lung_CT and X had a strong relation with tumor_T (38), possibly along with the abnormal head_CT.

A retrospective dynamic evolution analysis found that the incidence of new critical values was about 10%, the incidence of persistent critical values was about 50%, and most of the effective evolution paths tended to have no critical values for men and women. Also, for some specific critical values, persistence and variability existed. Physical examination aims to screen the early diseases and provide the intervention regimen, while the critical values are the most important. Our results showed that the incidence of new critical values (10%) is about 30 times as much as that of the malignant tumors (285.83/100,000), hinting that 1 in 30 with abnormal critical values was likely to develop malignant tumors. In addition, critical values in health checkup mainly include clinical critical values, major diseases, and their clues, acute, and chronic lesions, and abnormal examination results require timely handling, and the participants possibly adopted some effective ways to intervene in progress of critical value after being informed, such as the changes of healthy lifestyle, surgical treatment, or drug intervention; thus, these participants were not detected by the critical values in the next year (39, 40).

## Study Limitations

The present study is the first to analyzes the critical values in health checkup at a large physical examination center in China. The limitations for the present study are the inclusion of a short-term retrospective cohort (only from 2017 to 2019) and the lack of comprehensive follow-up information, making the accurate incidence between critical values in health checkup and a participant cancer outcome unclear. Thus, with the recent trends of perfecting the whole process quality control in health checkup, further research should be conducted to develop personalized intervention regimens, combined with critical values in health checkup in the big data environment.

## Conclusions

Health management becomes more and more important in controlling risk factors, designing prevention regimens, and further improving health quality, whereas critical value management in health checkup is a crucial step. This study conducts a cross-sectional and retrospective survey to analyze the prevalence, association relation, and dynamic evolution of critical values at a large physical examination center in Central China from 2017 to 2019. The findings of this study provide historical data supporting further research to explore health management decision-making at the population level and the prevention and treatment of various types of cancer and NCDs at the individual level.

## Data Availability Statement

The original contributions presented in the study are included in the article/supplementary material, further inquiries can be directed to the corresponding author.

## Ethics Statement

The studies involving human participants were reviewed and approved by the Institutional Review Board of the First Affiliated Hospital of Zhengzhou University (2018-KY-56). The patients/participants provided their written informed consent to participate in this study.

## Author Contributions

JC and SD designed the experiments. JC and ZW analyzed the data. YL, LW, TL, YD, and QQ provided the data. JC wrote the paper. SD and ZW reviewed and edited the paper. All authors contributed to the article and approved the submitted version.

## Conflict of Interest

The authors declare that the research was conducted in the absence of any commercial or financial relationships that could be construed as a potential conflict of interest.
